# Childhood Multiple Endocrine Neoplasia (MEN) Syndromes: Genetics, Clinical Heterogeneity and Modifying Genes

**DOI:** 10.3390/jcm13185510

**Published:** 2024-09-18

**Authors:** Francesca Lanzaro, Delia De Biasio, Francesco Giustino Cesaro, Emanuela Stampone, Immacolata Tartaglione, Maddalena Casale, Debora Bencivenga, Pierluigi Marzuillo, Domenico Roberti

**Affiliations:** 1 Department of Woman, Child and General and Specialized Surgery, University of Campania “Luigi Vanvitelli”, Via Luigi De Crecchio 2, 80138 Naples, Italy; 2 Department of Precision Medicine, University of Campania “Luigi Vanvitelli”, Via Luigi De Crecchio, 7, 80138 Naples, Italy

**Keywords:** MEN, cancer predisposition syndromes, modifying genes

## Abstract

Multiple endocrine neoplasia (MEN) syndromes are part of a spectrum of clinically well-defined tumor syndromes ultimately characterized by histologically similar tumors arising in patients and families with mutations in one of the following four genes: *MEN1*, *RET*, *CDKN1B*, and *MAX*. The high level of genetic and phenotypic heterogeneity has been linked to phenocopies and modifying genes, as well as unknown mechanisms that might be investigated in the future based on preclinical and translational considerations. MEN1, also known as Wermer’s syndrome (OMIM *131100), is an autosomal dominant syndrome codifying for the most frequent MEN syndrome showing high penetrance due to mutations in the MEN1 gene; nevertheless, clinical manifestations vary among patients in terms of tumor localization, age of onset, and clinical aggressiveness/severity, even within the same families. This has been linked to the effect of modifying genes, as described in the review. MEN 2-2b-4 and 5 also show remarkable clinical heterogeneity. The traditional view of genetically predisposing monogenic or multifactorial disorders is no longer valid, and mandates a change in scientific focus. Phenotypes are indeed rarely consistent across genetic backgrounds and environments. In the future, understanding factors and genetic variants that control cellular functions and the expression of disease genes should provide insights into fundamental disease processes, providing implications for counseling and therapeutic and prophylactic possibilities.

## 1. Introduction

Multiple endocrine neoplasia (MEN) is a rare group of cancer predisposition syndromes characterized by the occurrence of two or more endocrine gland tumors in an individual or related individuals in the same family. These disorders may be inherited as an autosomal dominant trait with high penetrance [1], or may occur sporadically [2,3]. They can arise at any age, and their symptoms may vary based on the involvement of different organs or glands. The scientific literature recognizes five different disorders (MEN1–5) identified by mutation in different genes and distinguished phenotypically by the development of synchronous or asynchronous tumors in specific endocrine glands [3,4,5,6].

Although they are dominantly inherited in monogenic diseases, disease phenotypes are unpredictable and differ even among members of the same family [7]. There is a lack of systematic analyses among the different genetic modifier disorders that this review tries to address, and it also tries to propose unexplored genetic linkages, based on preclinical and clinical findings, that might be worth investigating in patients affected by MENs.

Many other cancer predisposition syndromes, such as von Hippel–Lindau syndrome [8], Neuro-fibromatosis type 1 [9] and tuberous sclerosis [10], are linked to a higher incidence of endocrine and neuro-endocrine tumors, but for the sake of this review, the authors focused on primary MEN syndromes.

Multiple endocrine neoplasia type 1 (MEN1) syndrome (or Wermer’s syndrome) is caused by heterozygous loss-of-function mutations in the tumor suppressor gene *MEN1*. The main districts involved in this syndrome are parathyroid, pancreatic islets, anterior pituitary, and the gastrointestinal tract [2,3].

MEN2 syndrome occurs due to mutations in the *Rearranged during Transfection* (*RET*) proto-oncogene that are encoded by a tyrosine kinase receptor [11,12].

MEN2 is characterized by tumors in the multiglandular site. It includes two independent clinical phenotypes, MEN2A and MEN2B, depending on the glans involved (thyroid, parathyroid, and adrenal glands) [13]. Moreover, according to the latest American Thyroid Association (ATA) 2015 guidelines, familial medullary thyroid carcinoma (FMTC) is recognized as a variant of MEN2A with reduced penetrance of hyperparathyroidism and pheochromocytoma [12,14].

MEN4 is the most recent member of the MEN syndromes group; it was first described in 2006 as an autosomal dominant disorder due to a mutation in the *cyclin-dependent kinase inhibitor 1B* (*CDNK1B*) gene [15]. Because of its clinical presentation, MEN4 could be considered a variant of MEN1 [13], occurring at a higher average age [16]. Patients develop MEN1-associated tumors, including parathyroid tumors (approximately 80% of patients) [17] and anterior pituitary tumors that are typically smaller and less aggressive than MEN1, in association with tumors of the kidneys, adrenals and reproductive organs [15,18]. *CDNK1B* must therefore be considered a new susceptibility gene for cancer predisposition syndrome.

MEN5 is the rarest and newest MEN syndrome identified, and it is related to germline *MAX* mutations associated with pheochromocytomas, ganglioneuromas, neuroblastomas, pituitary neuroendocrine tumors, and parathyroid adenomas, as well as nonendocrine tumors such as chondrosarcoma and lung adenocarcinoma [19].

The high level of genetic and phenotypic heterogeneity is described in Table 1, and has been linked to the presence of phenocopies and modifying genes [20]. Updates are often given in the scientific literature.

This review will focus on the clinical features, genetics and epigenetics of the MEN syndromes.

## 2. MEN1

MEN1, also known as Wermer’s syndrome (OMIM *131100), is an autosomal dominant syndrome with a worldwide prevalence of 3–20 in 100,000 individuals and a high penetrance due to mutations in the *MEN1* gene [11,12,13,14].

The *MEN1* gene is located on chromosome 11 (11q13), consists of 10 exons, and encodes for a nuclear protein of approximately 610 amino acids, called “Menin”. Menin is a ubiquitin protein without intrinsic enzymatic properties, but it plays a crucial tumor-suppressor role, and contributes to epigenetic regulation and gene transcription through its interaction with chromatin-associated protein complexes and transcription factors [21]. Menin’s crystalline structure presents an interaction site, resembling a deep pocket, enabling interaction with over 50 proteins, taking part in multiple pathways and processes such as gene transcription, cell adhesion, cell cycle progression, cell division, cell motility, cell signaling, cytoskeletal structure, DNA repair, genomic stability and transcriptional regulation. Examples of proteins and enzymes that interact with the *MEN1* gene product include SMAD1/SMAD5 and SMAD 3, TGF-β, PPARα, PPARϒ, GFAP, Nm23H1 and others [22]. 

Mutations in the *MEN1* gene occur across its open reading frame, with over 1300 mutation sites identified leading to many amino acid changes [23].

These mutations exhibit autosomal dominant transmission and are distributed throughout the entire coding region without a significant hotspot. 

Approximately 50% of the mutations are insertions and deletions causing frameshifts (e.g., c.1546dupC (p.Arg516Profs∗15), 20% are missense mutations, 20% are nonsense mutations and about 7% involve defects in the splice site. Many of the nonsense mutations, insertions, frameshift deletions, and splice site mutations lead to truncations of the Menin protein product, resulting in loss of protein function. Notably, 10–20% of individuals affected by MEN 1 do not have mutations in the coding region of the MEN1 gene, suggesting that these patients may have mutations in the untranslated regions (UTRs) or promoter region, areas still under investigation [22]. Roughly 75% of MEN1 mutations are inactivating, a common feature of tumor suppressor genes [24]. Interestingly, somatic *MEN1* mutations are also detected in 30–40% of MEN1-like sporadic tumors [21].

Clinical features occur in 50% of affected individuals by the age of 20 years and in 95% by the age of 40 years [2,25]. MEN1 is characterized by varying combinations of more than 20 endocrine and nonendocrine tumors involving two or more endocrine glands, typically the parathyroid glands, pancreatic islets, and anterior pituitary. Other MEN1-associated tumors include thymus and lung neuroendocrine tumors (NETs), type 2 gastric NETs, adrenocortical tumors, pheochromocytomas, facial angiofibromas, collagenomas, meningiomas, ependymomas, lipomas, carcinoid tumors, breast cancer, and an increased risk of developing breast cancer in female patients [26,27,28,29,30,31]. Both familial and sporadic forms exist; the expression of the MEN1 phenotype is age-dependent, although features are rare before 5 years of age [25,32,33]. The pathogenesis of MEN1 is linked to the Knudson’s 2-hit hypothesis, where a somatic inactivation of a wild type allele leads to a loss of biological functions for the gene product [34]. MEN1-related tumors often occur due to a loss of heterozygosity at 11q13 [35], but other unconfirmed hypotheses have been proposed, such as a “negative feedback loop” between miR-24-1 and Menin that acts by silencing the expression of the second MEN1 wild-type allele through a post-transcriptional, reversible, epigenetic modality [36].

The diagnosis is indicated by the occurrence of two or more synchronous or metachronous typical tumors, or in patients with at least one endocrinopathy and a first-degree relative who is affected by one of these tumors (familial MEN 1). The identification of a germline MEN1 mutation provides a genetic diagnosis in symptomatic patients; however, genetic testing in asymptomatic relatives of MEN1-affected patients remains diagnostic [2]. Regardless, 5–25% of individuals are described as phenocopy, which means a clinical diagnosis of MEN1 without a genetic mutation in the *MEN1* gene [37,38].

The most common manifestation of MEN1 is primary hyperparathyroidism (PHPT), typically manifesting at around 20–25 years old, with 90% of patients developing PHPT at 50 years old. Children affected by MEN1 commonly develop PHPT after age 10 [3,39,40,41]. Males and females show the same prevalence [2,41]. Asymptomatic hypercalcemia is the most common initial manifestation; rarely, they manifest fatigue, depression, confusion, anorexia, nausea, vomiting, polyuria, polydipsia, constipation, bone pain or kidney stones, hypertension, and shortened QT interval. A recent retrospective analysis of the clinical manifestations of 48 patients with familial PHPT reported higher levels of serum PTH and calcium at diagnosis in MEN1 patient than in those with other syndromes. They further asserted that the first clinical manifestation in their MEN1 patients was gastroenteropatic neuroendocrine tumor and not PHPT, which progresses silently [42]. The malignant transformation of parathyroid tumors is uncommon, even if rare cases of parathyroid carcinoma have been reported in MEN1 patients, with only 21 cases reported in recent literature [43,44,45]. Independently, it has been also shown that mutations causing the disruption of the C-terminal end of parafibromin, encoded by the *CDC73* gene, are closely associated with the development of parathyroid tumors [46]. *CDC73* thus could represent a potential additional gene influencing the phenotypic spectrum of MEN syndromes, particularly regarding hyperparathyroidism associated with parathyroid gland neoplasms.

The second most frequent manifestation of adult MEN1 patients is pancreatic NETs [2]; however, it is the rarest in young patients. The onset is generally after the age of 40 in 70–80% of cases [13]. Pancreatic NETs can exhibit malignant behavior and include gastrinomas (40%), insulinoma, glucagonomas, non-functioning pancreatic polypeptide-secreting tumors, and vasoactive intestinal polypeptidomas (VIPomas) [2].

Gastrinomas, characterized by excessive gastric acid production and recurrent peptic ulcers, are usually located in the duodenum (three times more than in the pancreas) and occur in individuals older than 30 years old [2,32]. It is rare in children. In total, 50% of MEN1 patients clinically manifest as Zollinger–Ellison Syndrome (ZES), a syndrome characterized by gastric acid hypersecretion, peptic ulcer disease, upper-abdominal pain, gastroesophageal reflux, and secretory diarrhea. Diagnosis is established by identifying serum gastrin levels greater than 10 times the upper limit of normal [2,47]. Associated hypercalcemia, due to PHPT, may worsen the symptoms because of increases in gastrin secretion. Locoregional lymph node metastases are present in 70–80% of duodenal gastrinomas at diagnosis, with the presence of liver metastases (approximately 25–50%) being the most significant predictor of survival [2,47,48,49]. Pancreatic gastrinomas are more aggressive than duodenal; they more frequently involve liver metastasis, above all occurring in symptomatic and older patients, and are bigger than duodenal tumors. Hypergastinemia may alter iron status via a CCK2R-independent mechanism, as observed in both mutant mice and in human patients with MEN, and seems to be correlated positively with transferrin saturation [50]. Iron deregulation may result from a direct regulation or mutation in DMT-1, a duodenal divalent metal transporter implicated in rare forms of hereditary hypochromic microcytic anemias [51], potentially influencing tumor biology [52].

Insulinoma usually involves a tumor secreting pancreatic beta cells, clinically presenting with recurrent hypoglycemia episodes associated with inappropriately elevated insulin and c-peptide levels. Lesions are typically benign, and the incidence is around 10%, with onset occurring earlier than in sporadic cases [2].

Glucagonomas arise from alpha cells of the pancreas, and occur in less than 3% of adults with MEN1. About 80% are malignant and frequently occur with liver metastasis [2]. Recent advances have reported the presence of MEN1 mutation in 60% of sporadic glucagonomas [32]. Clinical signs of glucagon secretion include hyperglycemia, anorexia, anemia, diarrhea, glossitis, venous thrombosis and necrolytic migratory erythema.

About 3% of MEN1 patients develop a VIPoma during their life, typically presenting as a malignant tumor that is metastatic at the point of diagnosis. Tumors are often greater than 3 cm, and are characterized by WDHA syndromes: watery diarrhea, metabolic acidosis, hypokalemia and achlorhydia.

Non-functioning NETs occur in 55% of adult MEN1 cases [33,53], and they are usually detected incidentally during gastric endoscopy or ultrasound tests. Interestingly, data from an ultrasound screening program in young MEN1 carriers have provided evidence of the high prevalence of these tumors being clinically silenced during the second decadence (42%) [54]. Endoscopic ultrasound (EUS) examination is the most sensitive imaging procedure for the detection of small (≤10 mm) pancreatic endocrine tumors in asymptomatic individuals with MEN1 [55,56,57]. By age 50, the penetrance of these tumors in MEN-1-affected persons reaches 34%.

Pituitary adenomas are the third-most common tumors in adults with MEN1 (30 to 50% of individuals), and the second-most common in children (34%), occurring at 5 years old [39,58,59]. Up to 6.5% of sporadic pediatric prolactinomas are linked to undiagnosed MEN-1 [60].

Microduplications of chromosome Xq26.3 have been associated with a new disorder of X-linked acrogigantism (X-LAG) [61] and the development of mixed GH and/or prolactin adenomas, presenting in pediatric patients (before 5 years of age) [62]. Such variants, and mostly alterations, in the genes and genetic material involved in the microduplications, potentially representing a factor influencing the variability of the phenotypic spectrum of MEN1 syndrome and age of onset, could be investigated in pre-clinical models and patients with genetically defined MEN1 syndromes.

Adult pituitary adenomas are more frequent in females, but in children they are more prevalent in males [2]. Males in both age groups present more frequently with macroadenomas and more severe onset symptoms [39,63]. Clinical manifestations depend on the hormones secreted and the tumor size [33]. The malignant progression of these tumors is rare, and only a few reports have described metastatic or malignant pituitary adenoma [64,65,66]. Vergès et al. reported that 32% of these tumors are invasive [2,63]. Vasopressin has rarely been linked to MEN-related pituitary adenomas [67,68], although the gene promoter has been implicated in a potential relationship between the endocrine tumor syndrome observed in vasopressin-driven SV40 transgenic mice and familial MEN [69]. The specificity of vasopressin gene expression normally results from an interaction between several regulatory elements, some of which are absent in the hybrid oncogene, and mutations in this gene have been reported to cause autosomal dominant neurohypophyseal diabetes insipidus [70].

Macroadenomas may cause mass effects, such as visual field defects, headaches, and/or hypopituitarism. Prolactinomas are the most common pituitary lesion for all ages, with associated signs and symptoms including hypogonadism, infertility, and/or galactorrhea [2,33,39,58]. Somatotropinomas can cause acromegaly in adults and gigantism in children, while corticotropinomas secrete corticotropin autonomously, resulting in hypercortisolemia and Cushing disease. Thyroid-stimulating hormone (TSH)-secreting anterior pituitary adenomas cause signs and symptoms of hyperthyroidism.

Carcinoid tumors, such as thymic, bronchial, and type II gastric enterochromaffin-like (ECL) carcinoids, occur in 3–10% of individuals with MEN1. Thymic carcinoids are more prevalent in males, with a male:female ratio of 20:1 [2], and they are often aggressive, particularly in smokers, sometimes presenting with metastasis at the onset. Bronchial carcinoids occur usually in women, and are generally less aggressive, even if they can cause mass effects, metastasis, and recurrence after surgery.

As described before, the MEN1 phenotype varies between patients in terms of tumor localization, age of onset, and clinical aggressiveness, even between affected members within the same family. Additional co-segregating modifying factors, such as germline mutations in other genes, or epigenetic changes or post-translational protein modifications, likely contribute to the interfamilial variability of MEN 1 [18].

For example, the gene encoding for the aryl hydrocarbon receptor interacting protein (*AIP*) is located on 11q13, near the *MEN1* gene but approximately 3 mb away. Inactivating mutations and deletions in the *AIP* gene predispose individuals to low-penetrance pituitary adenomas. Concurrent deletions involving these genes may contribute to predisposition to MEN1 and pituitary adenoma [71], as has been already postulated for the pathogenesis of the brown fat tumor hibernoma [72]. Although no preclinical models or clinical direct evidence have been produced in the MEN1 setting, patients with particularly aggressive pituitary adenomas associated with MEN1 syndrome might be considered for either *AIP* gene analysis or expression and functional protein assessments, which could help explain the aggressive phenotype.

The aryl hydrocarbon receptor-interacting protein (AIP) is a relatively understudied HSP90 and HSC70 co-chaperone, consisting of 330 amino acids, with a potential role as both a tumor suppressor and an oncogene involved in the cAMP-phosphodiesterase pathway. Common *AIP* variants (AIPvar) include nonsense and missense mutations, deletions, insertions, splice-site and promoter mutations, and large deletions, most of which lead to a truncated protein or, less commonly, affect the tetratricopeptide repeat (*TPR*) domains or the C-terminal α-helix. Additionally, germline *AIP* variants may result in a loss of heterozygosity (LOH) at the *AIP* gene site in the 11q13 region, and significantly predispose individuals to pituitary adenomas [73], which present at a younger age with larger sizes and increased aggressiveness, challenging traditional treatment methods [74].

Another gene biologically linked to the MEN ontology functions is the vitamin D receptor (*VDR*) located on chromosome 12 (12q13.11). This is a nuclear receptor involved in the transcription of genes related to the signaling pathway of vitamin D, calcium, and phosphorus, as well as cellular proliferation processes and the control of the immune system. The best-known polymorphisms of *VDR* are Fokl (rs10735810), BsmI (rs1544410), Taql (rs731236) and Apal (rs7975232). These polymorphisms are associated with the development of cardiovascular alterations, rheumatic arthritis and metabolic bone diseases, type 2 diabetes, cancer, and autoimmune diseases [75].

Patients with inactivating mutations in the *VDR* gene exhibit end-organ resistance to calcitriol. In patients with MEN1, a decreased activation of VDR in the parathyroid glands is observed, potentially contributing to hyperactivity, hyperplasia, and adenoma [76].

Longuini et al. reported that the p27 c.326T>G (V109G) variant may act as a disease modifier in MEN1 syndrome cases associated with germline MEN1 mutations in a Brazilian cohort [77]. Subsequently, Circelli et al. confirmed that MEN1-related aggressive tumors, or other malignancies, have been shown to be more frequent in patients with the CDKN1B V109G variant. It is still not possible to clearly state whether this p27 variant behaves as a real modifying gene or not [78].

## 3. MEN2

Multiple endocrine neoplasia 2 syndrome has an estimated prevalence of 1 in 30,000 [13].

Mutations in the oncogene *RET* are associated with the onset of these endocrine neoplasms. The *RET* gene is located on chromosome 10 (10q11.2), composed of 21 exons, and encodes a receptor tyrosine kinase (RTK) protein. RET consists of a cysteine-rich extracellular domain, a transmembrane domain, and an intracellular domain with tyrosine kinase activity. Physiologically, RET receptors are activated by ligands belonging to the glial cell line-derived neurotrophic factor (GDNF) family, which bind to the GDNF family receptor alpha coreceptor (GFRα) [79]. The mutations primarily observed in *RET* and linked to these two rare neoplastic conditions are mainly missense variants leading to a ligand-independent constitutive activation of the RET receptor, the autophosphorylation of RET, and the aberrant stimulation of downstream signaling pathways [80]. A great number of somatic alterations have been found to confer the constitutive activation of its kinase activity, a causative factor in many cancer subtypes [81]. Although loss- versus gain-of-function alterations can act in a context-dependent manner to initiate or favor tumorigenesis, the genes involved in cancer predisposition syndromes have mostly been described as tumor suppressors, differently from the MEN2 etiology [82,83]. Not all RET mutations have equivalent clinical significance; indeed, it is possible to stratify the risk of MEN2 by genotype [40,84]. Therefore, even on the molecular level, different deregulations are emerging, impacting the molecular signaling to different degrees [80].

As indicated by the former concept, disease features frequently vary among carriers of an identical *RET* mutation, as well as among family members, diagnosed at different ages, with different aggressiveness, with or without associated pheochromocytoma (PHEO) and primary hyperparathyroidism (PHPT), suggesting the existence of other factors modifying the disease’s course [85].

MEN2A mutations were detected mainly in one of six cysteine residues of the RET extracellular domain, leading to disulfide-linked RET dimerization. The MEN2B mutations were found at methionine 918 (methionine 918 → threonine) or alanine 883 [86] in the kinase domain, and activated RET without dimerization. Specifically, 95% of *RET* mutations occur in the cysteine-rich extracellular domain, with 85% of these mutations located at codon Cys634. Approximately half of the mutations at codon Cys634 found in MEN2 are Cys634Arg (C634R) substitutions [87].

Typically, the clinical manifestations of MEN2 patients include medullary thyroid carcinoma (MTC) and pheochromocytoma (PHEO); the different combinations of the endocrine neoplasia with or without nonendocrine diseases give rise to the following independent phenotypes/diseases: MEN2A, MEN2B and FMTC (which is recognized as a variant of MEN2A). The severity and age of onset differ between the forms. The diagnosis of MEN2 is established in a proband who fulfills existing clinical diagnostic criteria (two or more classic endocrine manifestation) or by identification via the molecular genetic testing of a heterozygous germline gain-of-function variant in *RET*. The guidelines recommend that all patients affected by MTC receive routine genetic screening for RET mutations, because 25% of these are due to a genetic error [13,88,89]. *RET* germline mutations in exon 16 codon M918T are the most common pathogenic variants associated with MTC, occurring in approximately 95% of patients.

MEN2A, also known as Sipple syndrome, has an estimated prevalence of 1 in 36,000 to 1 in 200,000 live births (Table 1) [90], and is characterized by MTC, PHEO and PHPT.

MTC, typically the first manifestation of MEN2A, occurs in the third decade of life in more than 90% of individuals [2,91]. Due to familiar screening, the diagnosis is usually reached in asymptomatic individuals manifesting as a thyroid nodule; rarely, signs and symptoms, such as anterior neck lump, difficulty with breathing or swallowing, or hoarseness, may occur. In children, MTC may present before the age of 6, with some cases identified in children as young as 2 years of age [92]. Elevated calcitonin levels upon routine screening are also noted prior to the development of clinical symptoms. Diarrhea (the most frequent systemic symptom) occurs in affected individuals with a plasma calcitonin concentration of >10 ng/mL, and implies a poor prognosis. Interpreting serum calcitonin data is challenging; clinicians should be aware that calcitonin levels are markedly elevated in children under 3 years of age, and especially in those under 6 months old, and are higher in males compared with females. Finally, falsely high or low serum calcitonin levels might occur in a variety of clinical diseases other than MTC; clinicians should consider this possibility when these levels are disproportionate to the expected clinical findings [93].

PHEO and PHPT are usually found in adulthood, and the penetrance is influenced by the specific underlying *RET* mutation [93,94,95].

Pheochromocytoma affects up to 50% of adults MEN2A during the second decade of life; however, children as young as 8 years of age have been described with PHEO as well [96,97]. MEN2A-related PHEOs are usually bilateral and localized, and metastasis rarely occurs [93]. The elevated excretion of catecholamines and catecholamine metabolites (e.g., norepinephrine, epinephrine, metanephrine, and vanillylmandelic acid) in plasma or 24 h urine collections is diagnostic [93,98,99]. Clinical features may include headaches, flushing, diaphoresis, palpitations, tremors, nausea, and anxiety.

PHPT occurs in 10–30% of MEN2A patients and is generally mild, presenting as a benign adenoma or parathyroid hyperplasia [96,100]. The mean age of diagnosis is approximately 34 years; however, children as young as 2 years of age have been diagnosed [101]. MEN2A-PHPT is asymptomatic for a long time, and often, hypercalcemia is the first sign. The symptomatic form may clinically present with lethargy, depression, confusion, anorexia, constipation, nausea, vomiting, diuresis, hypercalciuria, kidney stones, increased bone resorption, and increased fracture risk due to hypercalcemia [101].

Cutaneous lichen amyloidosis (CLA) is a rare dermatologic manifestation of MEN2A that may present at young age prior to another sign [102,103]. It is an itchy skin lesion located in the interscapular region that improves with sun exposure and worsens with stress. Cutaneous lichen amyloidosis has been reported in some MEN 2A families affected by specific germline *RET* codon 634 mutations; this could lead to a novel genotype-phenotype spectrum in MEN 2A [104]. Hirschsprung disease is another condition that co-occurs with MEN2A [105].

Germline variants of the succinate dehydrogenase genes (*SDHB*, *SDHC*, *SDHD*, and the newly identified *SDH5* and *TMEM127* genes) have been suggested to play a role in MEN2a [106,107]. This intricate complex of proteins is indispensable for mitochondrial electron transport and ATP synthesis, comprising four subunits (A-D) with SDHAF2 and ensuring its structural integrity. Notably, subunits B, C, and D display robust associations with PCCs and PGLs [108]. The high prevalence of the SDHD^G12S^ variant in patients with multiple endocrine neoplasia type 2A raises questions about its role as a genetic modifier, but this proposal remains to be established [109]. Some hereditary paraganglioma syndromes manifest prominently only through the appearance of head and neck paragangliomas (HNPGL), arising from mutations impacting the succinate dehydrogenase (*SDH*) complex, and sometime they overlap with MEN2 [110].

The *TMEM127* gene, on the other hand, is a gene that acts mainly as a tumor suppressor; it encodes a transmembrane protein whose function remains elusive, and is frequently found mutated in pheochromocytomas and, to a lesser extent, in renal cancers. Tumors harboring *TMEM127* mutations exhibit heightened mTORC1 signaling, though the underlying mechanisms remain obscure. Pheochromocytomas carrying *TMEM127* mutations exhibit elevated levels of LAMTOR proteins [111], while generally, a loss of *TMEM127* drives RET-mediated tumor transformation, deregulating membrane dynamics [112]. It would be worth investigating *TMEM127* variants and protein levels in preclinical models or patients with MEN2 syndromes so as to assess the potential contributions of such variants to the clinical phenotypes.

FMTC is considered today as a clinical variant of MEN2A, and it can be diagnosed based on a set of specific criteria: more than 10 family members with MTC, multiple carriers or affected members over 50 years of age, and a sufficient medical history to exclude the presence of PHEO and PHPT, or the presence of at least four family members with MTC but without other manifestations of MEN2A [40,113,114].

MEN2B is instead rarer than MEN2A, with a prevalence of between 1 in 600,000 and 1 in 4,000,000 [115]. Patients are at high risk of developing MTC, which occurs much earlier than MEN2A and is the leading cause of death in MEN2B [116,117] and PHEO. Additional features of MEN2B include mucosal neuromas of the lips and tongue, distinctive facies with enlarged lips, ganglioneuromatosis of the gastrointestinal tract, eyelid eversion, prominent corneal nerves, scoliosis, and a marfanoid habitus. They may present with any nonspecific and precocious symptoms, such as failure to thrive, alacrimia and constipation. MTC typically occurs in early childhood in MEN2B as a thyroid nodule, and the variant shows an aggressive clinical progression [93]. MEN2B involves a 50% risk of developing PHEO, which may occur earlier than MEN2A and is more frequently bilateral [118,119,120]. About 40% of affected individuals show diffuse ganglioneuromatosis of the gastrointestinal tract. Associated symptoms include abdominal distention, megacolon, constipation, and diarrhea. In one study on 19 individuals with MEN2B, 84% reported gastrointestinal symptoms beginning in infancy or early childhood [121].

## 4. MEN4

MEN4 is a recently identified MEN syndrome that involves the same primary organs as MEN1, but is less common and tends to show a more indolent course. It is due to a mutation in the tumor-suppressor gene *CDKN1B* resulting in uncontrolled cell cycle progression [17,18]. 

*CDKN1B* (also referred to as p27) is located on chromosome 12p13 and has two coding exons, resulting in a 2.5 kb coding region for a nuclear protein, and one non-coding exon [17] encoding for p27, a member of the cyclin-dependent kinase inhibitor (CDKI) family that regulates cell cycle progression and arrest through its inhibitory function on various cyclin/CDK complexes, particularly the G1- to S-phase transition. It is an intrinsically unstructured protein [122] with several layers of post-translational regulation [123,124].

The protein p27 consists of 198 amino acids and contains a nuclear export signal (NES) at the N-terminal end and a nuclear localization signal (NLS) at the C-terminal end. The cell-cycle-inhibitory region is located between amino acids 25 and 93 and is necessary for binding to cyclin/CDK complexes. p27 exhibits different functions and interactions depending on its subcellular localization. Indeed, it moves from the nucleus to the cytoplasm, performing multiple functions including autophagy and cytokinesis, with a final impact on cell proliferation, survival, differentiation, motility and invasion, as well as transcriptional regulation and cytoskeleton organization. Additionally, it has been shown that the cytoplasmic interaction of the C-terminal domain of p27 with the protein stathmin, which destabilizes microtubules, interferes with microtubule dynamics and regulates cell migration; instead, the nuclear localization of p27 is necessary for its tumor-suppressive function in mice, and has been linked to the control of cell cycle progression through binding to cyclin/CDK complexes. The ability of p27 to interact with multiple partners, and, above all, its ability to participate in multiple signaling pathways with many diverse functions, makes it plausible that p27 could act both as an oncogene and as a tumor suppressor gene, depending on the context or interaction.

In most patients affected by MEN 4, mutations in the *CDKK1B* gene are missense mutations located within the coding sequence. Several mutations affecting the gene have been identified, such as the nucleotide substitutions c.678 C>T (p.P69L) and c.283 C>T (p.P95S), which disrupt the binding of p27 to GRB2 and CDK2; the in-frame deletions c.374_375delCT, c.59_77dup, and 371delCT influence the nuclear localization of p27. Furthermore, some germline mutations in *CDKN1B* have been identified that influence the expression of p27 by altering the UTRs, such as the deletions c.-456_-453delCCTT and c.-32_29delGAGA, which cause impairments in mRNA ribosome entry, on the one hand by elongating the upstream Open Reading Frame (uORF), and on the other by disrupting the mRNA secondary structure [125].

Hyperparathyroidism is the primary clinical manifestation of MEN4, followed by pituitary adenomas.

Primary hyperparathyroidism affects approximately 80% to 90% of patients with MEN4 [2]. PHPT presents later in MEN4 compared with MEN1, with a female predominance [126]. Hypercalcaemia is the most common initial manifestation. It may be asymptomatic, or less commonly has associated symptoms, including lethargy, depression, constipation, polyuria, polydipsia, renal stones, and osteoporosis.

Recurrence after parathyroidectomy has been reported in the literature only rarely, and after surgical glands resection, histology usually detects a parathyroid adenoma [16]. These data suggest a milder disease spectrum than MEN1 [127]. The indications for parathyroid surgery in MEN4 are the same as for MEN1, although there are no specific guidelines to date on the management of PHPT in MEN4.

The *CaSR* gene, encoded by the homonymous gene on chromosome 3, encodes for an important calcium-sensing receptor associated with G proteins that is crucial for regulating extracellular calcium homeostasis [128].

This gene has also been shown to play a role both as a tumor suppressor and as an oncogene, and its loss of function has been found in various neuroblastomas. Inactivating mutations in the *CaSR* gene, especially within the first 350 amino acids of the extracellular domain, lead to hypercalcemic disorders such as forms of primary hyperparathyroidism and familial hypocalciuric hypercalcemia (FHH) [129].

As shown by a study conducted on both patients with parathyroid adenomas and healthy controls, the expression of the *CaSR* gene in adenoma tissue is greatly reduced, as are the protein levels of p27 (10.1007/s12022-018-9524-9). Mutations affecting *CaSR* or epigenetic gene regulation, as well as post-translational modifications at the protein level, could be investigated in patients with MEN syndromes exhibiting phenotypic profiles mainly characterized by hyperparathyroidism, as this could indicate a gene that modifies the clinical picture.

The second most common manifestation of MEN4 is pituitary adenomas, emerging as both functional and nonfunctional tumors. The age of diagnosis varies from 30 to 79 years. Clinical manifestation depends on the size of the lesion and the secreted hormones (somatotropinoma, prolactinoma or corticotropinoma). Gigantism or acromegaly is reported in MEN4 [130,131,132], while prolactinomas are rare [16]. On the other hand, mutations in *CDKN1B* are very rare in cases of isolated pituitary adenomas, sporadic gigantism or acromegaly among the pediatric population [133,134]. A recent study found that 2.6% of patients with Cushing disease have the *CDKN1B* mutation [135]. The management of pituitary tumors in MEN4 is similar to that for other sporadic or familial cases—routine surveillance following the existing guidelines set out for other MENs.

Less frequently, MEN4 may be associated with neuroendocrinal tumors (NETs), tumors of the adrenals, kidneys, and reproductive organs. NETs in the context of MEN4 include functional or nonfunctional tumors located in the duodeno or stomach. The literature contains only few reports of tumors (NETs) in MEN4, the number of which is lower than that associated with MEN1.

There are no reported cases of insulinoma, vasoactive intestinal peptide-secreting tumor (VIPoma), glucagonoma, or ectopic adrenocorticotropic hormone (ACTH)-secreting neuroendocrine in patients affected by in MEN4 [17]. The methods of diagnosis and management of pNETs in MEN4 are similar to those for MEN1 [2].

Finally, to our knowledge, neuroendocrine cervical carcinoma, which has been reported frequently in MEN1, has been reported only in one patient affected by MEN4 [136]. Further, one study reported two nonsynonymous variants of *CDKN1B* implicated in primary ovarian insufficiency: p.V109G, a polymorphism, and p.I119T, a mutation [137].

## 5. MEN5

As already described above, recent advances in molecular analysis and familiar screening identified the pivotal role of new susceptibility genes in the pathogenesis of pheochromocytoma, such as *SDHA*, *TMEM127*, *MAX*, and *SDHAF2* [138,139]. Among these, heterozygous germline loss-of-function mutations in the tumor suppressor MYC-related factor X (*MAX*) gene were associated with hereditary pheochromocytoma–paraganglioma (PHEO/PGL) syndrome [140,141,142,143,144,145,146,147,148].

*MAX* is a gene located on chromosome 14, encoding for a protein of the same name, which acts as a tumor suppressor by participating in the gene transcription process. In particular, the MAX protein heterodimerizes with MAD factors, resulting in the repression of the oncogenic protein MYC. Inactivating mutations in the gene result in uncontrolled cell proliferation [149]. Mutations with loss of heterozygosity in the *MAX* gene have been correlated with the development of pituitary neuroendocrine tumors (PitNETs), pheochromocytomas, and paragangliomas. This condition has been identified as a new MEN syndrome, named MEN 5 [150]. The age of onset is in the second decade of life for PCC and in the third to fourth decades for PGL. Other associated conditions in MEN 5 include ganglioneuroma, neuroblastoma, primary hyperparathyroidism, lung cancer, pancreatic neuroendocrine tumor, renal cell carcinoma and renal oncocytoma [19].

Burnichon et al. performed an analysis of the *MAX* gene in 1694 patients with PHEO or PGL from different centers, highlighting that *MAX* variants were detected in 1.12% of these tumors [140]. Additionally, several case reports detected an association with the *MAX* germline mutation and the occurrence of endocrine and non-endocrine tumors in many families. Thus, the presence of *MAX* germline mutations linked to these clinical features led to the identification of Multiple endocrine neoplasia type 5 syndrome [19]. A recent systematic review reported that the most common *MAX*-associated tumor was unilateral or bilateral PHEO, and the main age of onset was 28.5 years old, followed by PGL (main age of onset 43 y.o.). Other features associated with the disease include pituitary adenoma, ganglioneuroma, neuroblastoma, pancreatic neuroendocrinal tumor, primary hyperparathyroidism, renal and lung cancer and erythrocytosis [151,152,153,154,155]. The pathogenic role of MAX mutations in these neoplasms is not completely clear, and future studies are needed to identify it along with the clinical phenotypes of these syndrome.

## 6. Conclusions

The best approaches to the determination and fine-tuning of phenotypes related to monogenic cancer predisposition syndromes with low penetrance are still not well established, although disease-specific genotype, epigenetic and environmental factors are certainly involved. Indeed, modifier genes must play a considerable role in the phenotypic heterogeneity of these disorders, linked to intrafamilial variations, incomplete penetrance, and varying disease severity.

MEN syndrome is an umbrella term used to describe a family of tumor syndromes ultimately characterized by histologically similar tumors arising in patients and families with mutations in one of four genes: *MEN1*, *RET*, *CDKN1B*, and *MAX*. Many other genes, as described in the review and summarized in Table 2, can explain (still only partially) the clinical heterogeneity and severity of the different clinical phenotypes, as well as the different levels of familiar penetrance. The traditional view of monogenic or multifactorial genetic predisposing disorders is no longer valid, and this mandates a shift in the scientific focus towards factors that modify gene expression, functions, and/or disease appearance and course. Phenotypes are indeed rarely consistent across genetic backgrounds and environments, and instead vary depending on allelic variants, unlinked genes, epigenetic factors, and environmental exposure. In the future, understanding factors and genetic variants that control cellular functions and the expressions of disease genes should provide insights into fundamental disease processes, and could provide implications for counseling patients while offering therapeutic and prophylactic possibilities.

## Figures and Tables

**Table 1:** Clinical and epidemiological features of different MEN syndromes.

Syndrome	Clinical Features	Incidence	Onset
MEN1	Parathyroid hyperplasia with primary hyperparathyroidism	>90%	20–25 y.o.
	Neuroendocrinal tumors: - Gastrinoma (40%); - Insulinoma; - Glucagonomas; - Non functional pancreatic polypeptide-secreting tumor (55%); - VIPoma.	30–80%	>40 y.o.
	Pituitary adenoma: - Nonfunctional tumor; - Prolactinoma; - GH-oma; - GH/PRL-oma; - ACTH-oma; - TSH-oma.	30–50% in adult and 34% in children	
	Other manifestations: pheochromocytomas, facial angiofibromas, collagenomas, meningiomas, ependymomas, lipomas, carcinoid tumors, breast cancer		
MEN2			
MEN2A	FMTC/Medullary thyroid carcinoma Or	100%	
	Medullary thyroid carcinoma associated with	100%	Third decade
	Pheochromocytoma	50%	Second decade
	Primary hyperparathyroidism	10–30%	34 y.o.
	Other: Cutaneous lichen amyloidosis		
MEN2B	Medullary thyroid carcinoma	100%	Earlier than MEN2A
	Pheochromocytoma	50%	
	Marfanoid habitus	75%	
	Other: Mucosal neuromas of the lips and tongue, ganglioneuromatosis of the gastrointestinal tract		
MEN4	Primary hyperparathyroidism	80–90%	
	Pituitary adenomas		30–79 y.o.
	Other: Neuroendocrinal tumors; tumors of the adrenals, kidneys, and testicular and cervical organs		
MEN5	Unilateral or bilateral pheochromocytoma		20 y.o
	Paraganglioma		30–40 y.o.

**Table 2:** Clinical heterogeneity of MEN syndromes: genetic background.

GENE	LOCUS	FUNCTION	MUTATION	PHENOTYPES
*MEN1*	Chromosome 11 (11q13)	Gene transcription and epigenetic regulation	Autosomal dominant: 50% of mutations are insertions and deletions causing frame shifts, 20% are missense mutations, 20% are nonsense mutations and approximately 7% are splice site defects	MEN 1 syndrome: mainly parathyroid glands, pancreatic islets, and anterior pituitary
*RET* (REarranged during Transfection)	Chromosome 10 (10q11.2)	Receptor tyrosine kinase protein; serves as a receptor for a series of proteins of the GDNF (glial cell line-derived neurotrophic factor) family	Substitutions; mainly 95% of RET mutations occur in the cysteine-rich extracellular domain (with 85% of these mutations located at codon Cys634) or in methionine 918 (methionine 918 → threonine) or alanine 883 in the kinase domain	MEN 2A: medullary thyroid carcinoma, hyperparathyroidism and pheochromocytoma MEN 2B: medullary thyroid carcinoma (more aggressive than MEN type A), pheochromocytoma and diffuse gastrointestinal ganglioneuromatosis FMTC: familiar medullary carcinoma.
*CDKN1B/p27*	Chromose 12 (12p13)	Inhibitor of cyclin-dependent kinase (CDKI), regulates cell cycle progression and arrest with inhibitory functions on various cyclin/CDK complexes, particularly at the transition from G1 to S phase, but many new functions have been discovered depending on its subcellular localization	Mainly missense mutations located within the coding sequence (for example: c.678 C>T (p.P69L) e c.283 C>T (p.P95S)) or germline mutations altering untranslated regions (UTRs)	Hyperparathyroidism and pituitary adenomas, with a more indolent course compared to MEN 1
*MAX* (Factor X associated with Myc)	Chromose 14 (14q23.3)	Tumor suppressor, transcription factor, which is a cofactor of the MYC proto-oncogene and plays an important role in the regulation of cell proliferation, differentiation and death	Inactivating mutations (truncating frameshift mutation, for example c.160C in exon 3 of the MYC-associated factor)	MEN 5: neuroendocrine tumors (PitNETs), pheochromocytomas and paragangliomas
*AIP* (Aryl hydrocarbon receptor-interacting protein)	Chromosome 11 (11q13)	Co-chaperone of HSP90 and HSC70 involved in the cAMP-phosphodiesterases pathway	Nonsense and missense mutations; deletions; insertions; splice-site and promoter mutations	Pituitary adenomas at a younger age, larger in size and more aggressive
*CaSR* (Calcium-sensing receptor)	Chromosome 3 (3q13.3-21)	Regulation of extracellular calcium homeostasis; apoptosis; cell proliferation	Inactivating mutations as missense mutations Hotspots cluster in exons 3, 4 and 7.	Primary hyperparathyroidism and familiar hypocalciuric hypercalcemia
*CDC73* (Parafibromin)	Chromosome 1 (1q31.2)	It participates in transcriptional processes important for chromatin remodeling, histone modification, initiation and elongation, and activates the wnt/ b-catenin and hedgehog signaling pathways	Mutations truncating the protein prematurely result in the loss of parafibromin function, especially with damage to the C-terminal	Parathyroid adenomas
*The VDR*	Chromosome 12 (12q13.11)	The transcription process of genes involved in the signaling pathway of vitamin D, calcium and phosphorus; cellular proliferation processes and the control of the immune system	Fokl (rs10735810), BsmI (rs1544410), Taql (rs731236) and Apal (rs7975232)	Parathyroid gland hyperplasia and adenomas; cardiovascular alterations, rheumatic arthritis and metabolic bone diseases, type 2 diabetes, cancer and autoimmune diseases
*SDHB*, *SDHC*, *SDHD*, *SDH5 e TMEM127*	Chromosome 1-2-11	SDH(B-C-D-5) encode the succinate dehydrogenase (SDH) comples, which are necessary for the mitochondrial electron transport chain and for the generation of ATP; TMEM127 is a tumor-suppressor gene that encodes a transmembrane protein of unknown function	Amino acid substitutions, truncating mutations, rearrangements, missense mutations, which all cause the inactivation of TMEM127 and SDH	Pheochromocytomas, renal cancers, gastrointestinal stromal tumor, papillary thyroid carcinoma and paragangliomas
*Xq26.3* (*GPR101*)	Chromosome X	An orphane G-protein coupled receptor, the role of which is not yet known but it is able to influence GH levels at the pituitary and hypothalamic levels	Microduplications	Development of mixed GH and/or prolactin adenomas
*AVP* (*vasopressin gene*)	Chromosome 20 (20p12)	Receptor V1a—vasoconstriction, gluconeogenesis, platelet aggregation, release of factor VIII and von Willebrand factor; Repector V1b—secretion of adrenocorticotropin (ACTH) in response to stress; V2—insertion of aquaporin-2 (AQP2) (channels for the passage of water)	Missense mutations c.173 G>C (p.Cys58Ser) e c.215 C>A (p.Ala72Glu)	Pituitary adenomas
*SLC11A2* (*DMT-1*)	Chromosome 12	It allows the uptake of iron in the kidneys and intestines and allows iron to be recovered from the recycling of endosomes	Splicing c.762 + 35A>G; substitution 223G>A	Tumors of the gastrointestinal system (colorectal cancer)

## Data Availability

This review aims to describe the clinical and genetic background of MEN syndrome patients, without a meta-analysis. We selected a great number of data from public databases (Pubmed, Medline, SCOPUS…) following a PRISMA flowchart. The data presented in this study are available on request from the corresponding authors.
